# High Prevalence of Non-typeable* Haemophilus influenzae* and *Haemophilus haemolyticus* Among Vaccinated Children with Community-Acquired Pneumonia in Vietnam

**DOI:** 10.1007/s44197-024-00195-8

**Published:** 2024-02-19

**Authors:** Xuan Duong Tran, Van Thuan Hoang, Thi Loi Dao, Pierre Marty, Philippe Gautret

**Affiliations:** 1 Thai Binh University of Medicine and Pharmacy, Thai Binh, Vietnam; 2 IHU-Méditerranée Infection, Marseille, France; 3 Aix Marseille Univ, IRD, AP-HM, SSA, VITROME, Marseille, France; 4 Université Côte d’Azur, Inserm, C3M, Nice Cedex 3, France; 5 Parasitologie-Mycologie, Centre Hospitalier Universitaire L’Archet, Nice Cedex 3, France; 6 Institut Hospitalo-Universitaire Méditerranée Infection, 19-21 Boulevard Jean Moulin, 13385 Marseille Cedex 05, France

**Keywords:** *H. influenzae*, *H. haemolyticus*, Children, Community-acquired pneumonia

## Abstract

Among 467 children under five hospitalized with community-acquired pneumonia, the prevalence of *Haemophilus influenzae* or *Haemophilus haemolyticus* was 60.8%, all cases were non-typable *H. influenzae* (NTHi) or *H. haemolyticus*. NTHi/*H. haemolyticus* PCR detection was associated with about twice the risk for severe disease. The results highlight the need for increased awareness and research efforts to investigate the role of NTHi/*H. haemolyticus* in severe CAP among children.

**Supplementary Information:**

The online version contains supplementary material available at 10.1007/s44197-024-00195-8.

## Introduction

Community-acquired pneumonia (CAP) is defined clinically as “the presence of signs and symptoms of pneumonia with a cough and/or difficulty breathing and/or fever and tachypnoea or chest indrawing (retraction), and an abnormal lung examination in a previously healthy child due to an infection which has been acquired outside hospital” [1]. CAP is a primary cause of morbidity and mortality in children under 5 years worldwide, particularly in low- and middle-income countries [2]. In 2019, pneumonia accounted for 14% of all deaths in children under 5 years and 22% of all deaths in children aged 1–5 years worldwide [3]. The Sub-Saharan Africa region had the highest proportion of CAP-related deaths, followed by South Asia, Latin America, and the Caribbean [3]. A recent study revealed that the overall incidence of pneumonia-related hospitalization was 16/10,000 children/year. However, this rate varied depending on the age group with the highest incidence being in children under 2 years of age [4].

The etiology of pneumonia among children is very variable. It depends on the age of patients, disease severity, and geographical factors [3, 4]. Several previous studies showed that *Haemophilus influenzae* was one of the most frequent pathogens detected in children with CAP, as described in a recent review [3]. Following the introduction of *H. influenzae* type b (Hib)-conjugate vaccines, the number of cases of invasive Hib infections, including bacteremic pneumonia, has declined wherever the vaccine has been implemented [5]. While it is well-known that Hib can cause serious infections such as meningitis, sepsis, and pneumonia, there is a growing concern regarding the increasing prevalence of non-typable *H. influenzae* (NTHi) among children with pneumonia [5].

In Vietnam, Hib vaccine was introduced in the national expanded immunization program since June 2010. The epidemiology of *H. influenzae* strains is known to have change due to the effects of vaccines [5]; however, this has not been studied in Vietnam yet. Therefore, we conducted this work to identify *H. influenzae* serotypes among children hospitalized with CAP in a Vietnamese hospital from July 2020 to June 2021.

## Methods

This cross-sectional study was conducted between 1 July 2020 and 30 June 2021 at the Thai Binh Paediatric Hospital, a tertiary hospital in northern Vietnam. Patients aged 5 years or under, who were hospitalised in the respiratory medicine ward with community-acquired pneumonia were enrolled in this study. Children over the age of five or those with pneumonia occurring after 48 h of hospitalisation were excluded from this study. The following data were collected from the central computer of the hospital: age, sex, clinical features, laboratory findings, and chest X-ray results.

Nasopharyngeal swabs were collected for each patient and then transferred to Sigma-Transwab® medium by a nurse in a standardised way. The samples were kept at room temperature before transfer to the laboratory department within one hour and stored at -80 °C. The samples were then transferred to Marseille at -20 °C on dry ice before subsequent investigation. Thermo Scientific KingFisher Flex extracted DNA and RNA from nasopharyngeal samples using the Kit NucleoMag^®^ Dx Pathogen (MACHEREY–NAGEL, Germany) according to the manufacturer’s instructions. The extracted products were tested by RT-PCR according to the manufacturer’s recommendations. The *SHD* gene (*SHD*-F1: 5′-GCGGCGAGATATTGACCTGT-3', *SHD*-R1: 5′-GCAGTGGYGGTATGGCAAAA-3′, *SHD*-Pr: 6FAM-TGAATTTTTAAAGGCDRCCACAACGGC-TAMRA) was used to screen all positive samples for *H. influenzae/H. haemolyticus* using a quantitative real-time PCR method with sensitivity and specificity was 100% and 100%, respectively (Supplementary data 1). The method, however does not allow distinguishing between *H. influenzae* and *H. heamolyticus*. Negative controls (PCR mix) and positive controls (DNA or RNA for bacterial or viral strain) were added to validate each RT-PCR test. The results were treated by CFX manager Software version 3.1 following the manufacturer’s recommendations. A sample was considered to be positive when a cyclic threshold value was equal to or less than 35.. *H. influenzae/H. haemolylitus*-positive specimens were subsequently serotyped using a real-time PCR method as previously described [6].

## Results

A total of 467 children with CAP were included. The results showed that *H. influenzae/H. haemolyticus* was detected with a prevalence of 60.8% (284/467). Interestingly, all cases were due to NTHi/*H. haemolyticus*. In NTHi/*H. haemolyticus* positive patients, the median was 14 months (interquartile = 5–27 months). Most patients were male (63.0%). A majority of them (89.8%) were fully vaccinated by their age according to the Vietnamese program [7]. Most common symptoms were cough (97.5%), tachypnea (87.7%), rhinorrhea (72.5%), wheezing (56.3%) and stridor when calm (36.6%). A proportion of 51.4% of patients were febrile and 14.8% had oxygen saturation < 90% in room air. Radiological findings showed that 38.7% of children had bilateral diffuse interstitial infiltrates. A proportion of 2.8% of patients had white blood count < 5 G/L and 21.5% had white blood count > 15 G/L, while 39.4% had CRP levels ≥ 50 mg/dL (Table 1).

**Table 1:** Clinical and paraclinical features at admission of patients positive with *H. influenza/H. haemolyticus* by real-time PCR (*N* = 284)

Characteristics	*n* (%)
Symptoms and signs	
Fever	146 (51.4)
Cough	277 (97.5)
Tachypnoea	249 (87.7)
Rhinorrhoea	206 (72.5)
Wheezing	160 (56.3)
Stridor when calm	104 (36.6)
Grunting	72 (25.4)
Chest indrawing	27 (9.5)
Persistent vomiting	9 (3.2)
Inability to drink or breastfeed	4 (1.4)
Severe respiratory distress	2 (0.7)
Central cyanosis	1 (0.4)
Oxygen saturation < 90% in room air	42 (14.8)
Abnormal pulmonary auscultation	284 (100)
Chest X-ray, laboratory, and microbiological investigation at admission in TBPH	
Chest X-ray	
Normal	4 (1.4)
Bronchial wall thickening	110 (59.9)
Bilateral diffuse interstitial infiltrates	170 (38.7)
White blood cell count (G/L)	
< 5	8 (2.8)
5–15	215 (75.7)
> 15	61 (21.5)
CRP levels (mg/dL)	
< 50	172 (60.6)
≥ 50	112 (39.4)

According to the guidelines of the Pediatric Infectious Diseases Society, of the Infectious Diseases Society of America and of the British Thoracic Society [8, 9], 88 (31.0%) cases were classified as severe pneumonia. Of note, NTHi/*H. haemolyticus* PCR detection was associated with about twice the odds for severe disease (adjusted OR = 1.66, *P* value = 0.04) (The multivariate analysis was performed using logistic regression by Stata v.17.0, after adjusting with age, gender, vaccination status, familial factors (presence of smokers and persons with acute cough at home, and laboratory findings).

## Discussion

NTHi is a widely recognized cause of lower respiratory tract infections in adults, especially those with chronic obstructive pulmonary disease [5]. According to recent studies, NTHi has become one of the most commonly isolated bacteria in respiratory tract infections in children, especially those under the age of five [5]. This is particularly concerning given that NTHi has been associated with a wide range of respiratory tract infections, including acute otitis media, sinusitis, pharyngitis, bronchitis, and pneumonia [10]. However, studies on the importance and role of NTHi in pneumonia in children are limited [5]. Despite its high prevalence, NTHi is often overlooked as a causative agent of respiratory tract infections, and there is a lack of awareness among healthcare providers regarding the importance of its detection and treatment. This may be explained by the difficulty in obtaining samples to determine the cause of lower respiratory tract infections in children. On the other hand, diagnostic panels for the responsive pathogens of respiratory tract infections using molecular biology methods only include Hib but not NTHi. Although data are limited, there seems to be little doubt that NTHi is responsible for a small proportion of pediatric pneumonia cases [5]. This rate may be higher in developing countries, causing a greater burden of disease on public health. The incidence and mortality rate of pneumonia in low-income countries is ten times higher than in developed countries [3, 5]. This perhaps reflects the underlying problems of poverty, malnutrition, overcrowding and inadequate health care in developing countries. In these countries, the microbiological diagnosis capacity is also limited due to lack of equipment. Recent studies have shown a small but steady increase in the incidence of invasive NTHi infection after routine vaccination against Hib [10–12]. Introduction of the Hib vaccine into immunisation programmes led to a rapid and sustained reduction in Hib disease across all age groups. As Hib vaccination reduces pharyngeal carriage, other *H. influenzae* strains could take its place and, cause high proportion of NTHi carriage. Therefore, the development of new and sensitive PCR technology will be invaluable in understanding the etiology and pathology of respiratory infections in children and may enable researchers to determine whether NTHi strains exist on mucosal surfaces and their relationship to invasive disease.

*H. haemolyticus* may account for 27% of apparent *H. haemophilus* strains identified by standard methods from nasopharyngeal isolates in the US [13]. It is considered to be a commensal with limited pathogenicity [14].

Our study has some limitations. The highly sensitive nature of RT-PCR makes it possible to detect the remaining material of dead together with active pathogens. The presence of NTHi/*H. haemolyticus.* in the nasopharynx does not necessarily mean it is the cause of CAP. NTHi/*H. haemolyticus* can be carried asymptomatically in the nasopharynx and it is, therefore, hard to demonstrate that NTHi/*H. haemolyticus* were the definitive cause of CAP. In addition, because sampling technique was not standardized and sampling was done at different stages of the disease depending on the time between onset of symptoms and children entry at hospital, we were not able to evaluate the viral or bacterial load of NTHi/*H. haemolyticus* detected by PCR. Another limitation of our study is the lack of a control group to assess the magnitude of healthy carriage of NTHi/*H. haemolyticus* Finally, distinction between NTHi and *H. haemolyticus* was not performed in our study, because no single target or current molecular methodology has been identified that can accurately identify all *H. influenzae* or *H. haemolyticus* strains [15]. Further studies using whole genome sequencing could be of interest.

In conclusion, the high rate of NTHi/*H. haemophilus* colonization among children with CAP and its association with severe disease highlights the need for increased awareness and research efforts in this area. While the Hib vaccine has been highly effective in preventing Hib infections, there is a pressing need for new vaccines that can provide protection against NTHi infections. Healthcare providers should be aware of the prevalence of NTHi and ensure appropriate detection and treatment, including judicious use of antibiotics.

### Supplementary Information

Below is the link to the electronic supplementary material.

Supplementary file1 (DOCX 19 KB)

## Data Availability

The data that support the findings of this study are available from the corresponding author, [PG], upon reasonable request.
