# Supplementary material for: High Prevalence of Non-typeable Haemophilus influenzae and Haemophilus haemolyticus Among Vaccinated Children with Community-Acquired Pneumonia in Vietnam
Source: J Epidemiol Glob Health. 2024 Feb 19;14(2):498–501. doi: 10.1007/s44197-024-00195-8 (PMC11176107; doi:10.1007/s44197-024-00195-8)
Supplement: Supplementary file 1 — Supplementary file1 (DOCX 19 KB) [file 44197_2024_195_MOESM1_ESM.docx]

**Supplementary document**

Comparison of established diagnostic methodologies and a SHD Real-Time PCR Assay for specific detection of *Haemophilus influenzae* isolates

The SHD gene (SHD-F1: 5'-GCGGCGAGATATTGACCTGT-3', SHD-R1: 5'-GCAGTGGYGGTATGGCAAAA-3', SHD-Pr: 6FAM-TGAATTTTTAAAGGCDRCCACAACGGC-TAMRA) was used to screen isolates of *H. influenzae, H. haemolyticus* and *H. parahaemolyticus, H. parainfluenzae, H. pittmaniae and H. sputorum* using a quantitative real-time PCR method. Well-characterized *Haemophilus* isolates previously identified by culture and matrix-assisted laser desorption ionization–time of flight mass spectrometry (MALDI-TOF MS) were obtained from the Institut Méditerranée Infection culture collection. A sample was considered to be positive when a cyclic threshold value was equal to or less than 35. All real-time PCRs were performed using a 7900HT-thermocycler (Applied Biosystems) and QuantiTect-Probe PCR Kit (QIAGEN, Courtabeuf, France) according to the manufacturer’s recommendations.

Results are presented in Table 1. Of the five *H. influenzae* isolates, five (100%) resulted positive by PCR, including when the DNA was diluted to 1/100. Of the four *H. haemolyticus* isolates 3 (75%) resulted positive by PCR, including when the DNA was diluted to 1/100. Of the eight other *Haemophilus* species tested, eight (100%) resulted negative when the DNA was diluted to 1/10.

Supplementary Table 1. PCR results of 17 isolates of *Haemophilus* spp., according to dilution.

| Isolate | Reference number | Ct | Ct 1/10 | Ct 1/100 |
| --- | --- | --- | --- | --- |
| *Haemophilus influenzae* | CSURP3559 | 29.08 | 31.64 | 34.00 |
| *Haemophilus influenzae* | CSURQ4091 | 9.77 | 13.72 | 17.43 |
| *Haemophilus influenzae* | CSURQ7843 | 9.23 | 13.42 | 16.64 |
| *Haemophilus influenzae* | CSURQ8269 | 11.66 | 15.42 | 18.62 |
| *Haemophilus influenzae type B* | CSURQ1821 | 11.34 | 15.32 | 18.59 |
| *Haemophilus haemolyticus* | CSURQ2070 | Negative | Negative | Negative |
| *Haemophilus haemolyticus* | CSURQ7663 | 11.75 | 15.94 | 19.36 |
| *Haemophilus haemolyticus* | CSURQP3472 | 20.01 | 24.05 | 27.1 |
| *Haemophilus haemolyticus* | CSURQ3869 | 16.07 | 20.04 | 23.97 |
| *Haemophilus parahaemolyticus* | CSURP7676 | Negative | Negative | Negative |
| *Haemophilus parahaemolyticus* | CSURP7683 | 34.06 | Negative | Negative |
| *Haemophilus parainfluenzae* | CSURP0991 | 31.47 | 35.71 | 39.02 |
| *Haemophilus parainfluenzae* | CSURP7511 | Negative | Negative | Negative |
| *Haemophilus parainfluenzae* | CSURP7513 | 31.74 | 39.02 | Negative |
| *Haemophilus pittmaniae* | CSURP9827 | 34.56 | 38.71 | Negative |
| *Haemophilus sputorum* | CSURP7341 | 36.94 | 38.32 | Negative |
| *Haemophilus sputorum* | CSURQ0127 | 32.68 | 36.54 | 37.39 |
